# Conducting non-commercial international clinical trials: the ICR-CTSU experience

**DOI:** 10.1186/s13063-017-2176-0

**Published:** 2017-09-26

**Authors:** Lisa Fox, Christy Toms, Sarah Kernaghan, Claire Snowdon, Judith M. Bliss

**Affiliations:** The Institute of Cancer Research Clinical Trials and Statistics Unit, ICR Clinical Trials and Statistics Unit (ICR-CTSU), 15 Cotswold Road, Sutton, Surrey SM2 5NG UK

**Keywords:** Clinical trial, International conduct, Academic trials, Rare cancers, Molecular subtypes

## Abstract

**Background:**

Academic clinical trials play a fundamental role in the development of new treatments, the repurposing of existing treatments and in addressing areas of unmet clinical need. With cancer treatments increasingly targeted at molecular subtypes, and with priority placed on developing new treatments for rare tumour types, the need for international trial participation to access sufficient patient numbers for successful trial conduct is growing. However, lack of harmonisation of international legal, ethical and financial systems can make this challenging and the cost and effort of conducting trials internationally can be considered prohibitive, particularly where the sample size is comparatively small.

**Methods:**

The Institute of Cancer Research – Clinical Trials and Statistics Unit (ICR-CTSU) is a UK-based academic clinical trials unit that specialises in the design, conduct and analysis of clinical trials of cancer treatments with an expanding portfolio of trials in molecular subtypes of breast and urological cancers and in other rare cancer types. Implementing appropriate mechanisms to enable international participation has therefore been imperative. In this article, we explain how we have approached the challenges involved and describe examples of successful international trial conduct, achieved through robust collaborations with academic and industry partners.

**Conclusion:**

Conducting academic trials internationally is challenging but can and should be achieved through appropriate governance mechanisms and strong collaborations.

## Background

In the era of precision medicine and with priority placed on developing new treatments for rare cancers, the need for international participation in academic clinical trials is increasing. However, lack of harmonisation of international legal, ethical and financial systems can make this challenging and the cost and effort of conducting trials internationally can be considered prohibitive, particularly where the sample size is comparatively small [1–5]. In this article we describe the progress, success and challenges faced by a UK-based, academic clinical trials unit, The Institute of Cancer Research – Clinical Trials and Statistics Unit (ICR-CTSU) to manage academically sponsored non-commercial international clinical trials.

## Main text

### Academic clinical trials units

There is no doubt that academic clinical trials make an important contribution to improvements in outcomes for cancer patients [6, 7]. ICR-CTSU is a UK university-based clinical trials unit that specialises in the design, conduct and analysis of academically sponsored cancer clinical trials. ICR-CTSU is a UK Clinical Research Collaborative registered clinical trials unit [8] and one of 15 units recognised by the National Cancer Research Institute for professional specialism in the development and delivery of cancer trials [9]. Comprising approximately 90 staff, ICR-CTSU provides academic leadership in trials, providing expert advice on scientific methods (statistical design and analysis) and also information governance, good clinical practice and data linkage. ICR-CTSU is responsible for all aspects of trial conduct including securing trial funding, protocol development, obtaining regulatory and ethics approvals, site selection and initiation, monitoring, pharmacovigilance and data analysis. ICR-CTSU receives core funding from the UK cancer charity, Cancer Research UK, which provides support to core senior multidisciplinary staff. Individual trial teams (trial managers, data managers, statisticians, programmers) and research procedures (trials unit and participating site research costs) are funded by charity and government trial-specific project grants and educational grants from industry.

### Sponsorship

Initial considerations around ICR-CTSU leadership of international trials focused on models of sponsorship. Whilst ICR traditionally favoured co-sponsorship, with allocation of sponsor responsibilities between ICR and the host institution of the chief investigator, this model was not widely recognised outside of the UK. This fundamental issue was resolved by ICR defining a framework for sole sponsorship of international clinical trials. In order to accept the role of international sponsor, ICR requires that defined criteria are met, including that there is a full justification for opening the trial internationally; the trial is already open and recruiting in the UK; national insurance arrangements are confirmed and ICR-CTSU must work with a collaborative group or lead site in each country who are willing to take on certain delegated national responsibilities.

### Country selection

The number of countries participating in an international trial has a considerable resource impact. Therefore, the decision of how many and which countries will participate is important. A small number of carefully selected countries are usually approached initially to participate. Factors affecting the choice of country include access to sufficient patient numbers, availability of clinical leadership and trials infrastructure, no known legal barriers, previous successful collaborations between the national collaborative group and ICR-CTSU and a proven track record of delivering multicentre international trials.

### Delegating country-specific sponsor responsibilities

The first task in each new proposed country is to identify a suitable collaborative group and confirm they can perform the required national delegated responsibilities. Although a seemingly simple task, this can take considerable time, from 6 months to in excess of 2 years, and, in some instances, the study is not able to proceed because agreement cannot be reached on all required responsibilities.

There are various reasons for the lengthy negotiations. For example, the standard scientific review process is often a two-stage process of ‘outline’ followed by ‘full’ application, where each application is reviewed at a formal scheduled meeting. Once scientific approval is in place, extensive operational negotiations with a different team often follow to establish local regulatory requirements and ensure the group can meet sponsor specifications for trial management and oversight.

The structure, level of resource, funding opportunities and operational experience of each collaborative group varies widely meaning a unique model of collaboration is often required for each country, and this takes time to establish and define contractually.

When trying to set up a trial in one country, following lengthy discussions around the collaboration model it became apparent that the collaborative group did not have the infrastructure to support certain delegated tasks such as on-site monitoring, so an alternative collaborative group had to be approached and the review process started again. In another country, although the collaborative group was able to take on some responsibilities, a contract research organisation (CRO) had to be appointed to take on others.

Although planning discussions are often lengthy, our experience is that they result in successful and efficient collaborations. Delegation of country-specific sponsor responsibilities ensures the collaborative group is responsible for all tasks that require local language, knowledge and experience, such as national ethical and regulatory submissions and approvals, country-specific safety reporting and local on-site monitoring.

### Insurance

In almost all instances, the most extensive discussions with the international collaborative groups relate to determining the national requirements for clinical trials insurance and arranging for additional country-specific cover to be put in place. Whilst in the UK a national indemnity scheme for clinical negligence claims exists, professional indemnity arrangements for clinical research may not be as clearly defined in other countries. A separate clinical trial insurance policy (in addition to the no-fault policy required by the EU Directive) is often required in third (non-EU) countries, and as the academic collaborative groups often already have this in place, the set-up process can be more straightforward and considerably faster outside of the EU.

### Collaborations with North America

UK sponsorship of clinical trials in the US remains difficult due to the differing regulatory, data protection and insurance requirements [10, 11]. For a UK academic sponsor, the additional costs of requisite legal consultancy and insurance for US participation can be prohibitive. EU concerns regarding data protection safeguards in the US exist making transfer of patient level data from the EU to the US extremely challenging. This does not have to prevent UK-led non-commercial trials running in the US and indeed ICR-CTSU currently collaborates with a US academic group to run the same trial in the UK and US, with parallel protocols. Separate databases exist for UK and US patients but the data are pooled for analysis in the UK and overseen by a single independent data monitoring committee. Whilst this circumvents the regulatory hurdles, it provides its own complexities in terms of consistency of trial conduct and data collection so robust processes for oversight and control via global oversight committees, comprehensive agreements and clear operating procedures are required.

### Funding international clinical trials

The source of national funding is also a point for extensive discussion. ICR-CTSU central trial management costs are usually met by charitable grant funding, but such funding may not be sufficient to support international trial management costs. As such, each national collaborative group may be required to secure funding for their local activities and if applicable, the responsibility for this is delegated to the national collaborative group. Delegation of this task allows the national group with expert local knowledge to define the level of local funding required and seek the most appropriate source. Funding may come from varying sources including the core funding of the collaborative group, national funding bodies and pharmaceutical market companies where relevant. The relative cost of running a trial may vary considerably between countries. The UK makes a distinction between commercial and non-commercial trials with an associated differential in costing and infrastructure support whilst many countries do not. There may also be added complexities in terms of good clinical practice (GCP) expectations; whilst in the UK non-commercial trials may be run according to the principles of GCP (as per Schedule 1 of the UK Medicines for Human Use (Clinical Trials) Regulations 2004) many countries mandate resource-intensive International Conference on Harmonisation (ICH)-GCP, which may increase local trial management costs (e.g. requiring 100% source data verification during on-site monitoring).

### Working with industry

Lack of sufficient funding is a recognised significant barrier to global academic cancer research [12]. Pharmaceutical companies can offer funding to an order of magnitude that enables international participation, something that many charity and government grants are unable to do. Academically sponsored trials are frequently conducted in collaboration with industry partners, and in these cases, the pharmaceutical company can offer not just grant funding, but assistance in setting up international participation. This can be in the form of market companies offering funding to support local trial management costs and in the planning and delivery of international drug distribution, including advising the sponsor on local drug labelling and import requirements. Local market companies can also provide support in terms of local language, regulatory knowledge and country-specific operational expertise.

### Our success story

The path of ICR-CTSU’s international trial management has by no means been straightforward but has been forged on strong and numerous collaborations. The CASPS trial of cediranib in patients with alveolar soft part sarcoma (ISRCTN63733470) closed to recruitment in July 2016, after reaching the required sample size of just 48 patients over a period of 5 years. Alveolar soft part sarcoma is rare, with an estimated incidence of only 15 cases per year in the UK. International participation in this trial was therefore imperative and has been a result of extensive, numerous and complex collaborations. To deliver the CASPS trial we received funding, support and advice from a UK cancer charity, the pharmaceutical company partner, a drug distribution company, international academic groups, their respective trials units and CROs, insurance brokers, contracts teams and lawyers. ICR, as trial sponsor, contracted directly with the pharmaceutical partner, the global drug distribution company, UK participating sites and the international collaborative groups. The collaborative groups obtained funding for local trial conduct and contracted with participating sites in their countries. A single global protocol existed with country-specific requirements for conduct detailed in supporting trial guidance notes to control the number of amendments. Country-specific approvals were obtained by the relevant collaborative group with support from ICR-CTSU to ensure consistency of applications. Trial operating procedures, for example for trial monitoring, were developed in collaboration with each collaborative group to allow for country-specific requirements. Collaborative groups provided trial approvals, monitoring reports and progress reports to ICR-CTSU to allow sponsor oversight globally throughout the life of the trial. This approach ensured clear lines of communication existed and trial conduct was consistent across participating countries. CASPS is the first international randomised trial to be conducted successfully in this rare disease.

### International trial collaboration recommendations

Table 1

**Table 1:** Recommendations based on the ICR-CTSU experience

Operational area	Challenges	Recommendations
Sponsorship	Co-sponsorship not currently recognised outside UK.	Institution to define requirements for sole sponsorship (NB. Co-sponsorship to be recognised in new EU regulation No 536/2014).
Country selection	Resource requirements increase with each participating country.	Careful consideration of the minimum number of countries required and choices based on previous successful collaboration.
Delegation of country-specific sponsor responsibilities	Not all groups are willing or able to take on the required country-specific sponsor responsibilities.	Define detailed, accurate and clear country-specific responsibilities early in negotiations.
Insurance	Country-specific insurance requirements are complex and expensive.	Work closely with specialist insurance broker with relevant experience.
Working with North America	Regulatory, data protection and insurance requirements can be prohibitive.	Seek alternative models of collaboration to sponsoring a trial in the US e.g. run parallel trials with the same protocol and database.
Funding	Central funding is not always sufficient to cover local costs.	Highlight possible funding shortfalls to collaborative groups early in discussions and ensure all funding requirements can be met within the proposed study timelines.

## Conclusion

Conducting international trials can be challenging for academic sponsors but can be achieved through appropriate governance arrangements, funding mechanisms and strong collaborations with international collaborative trials groups, and industry where applicable. CASPS is an excellent example of how such collaborations have helped to deliver an academic trial internationally. In trials of rare cancers where the sample size is comparatively small, although the required investment can seem disproportionate and difficult to justify, it is essential if progress is to be made in these areas of unmet clinical need.

## Abbreviations

CRO: Contract research organisation
GCP: Good clinical practice
ICH-GCP: International conference on harmonisation good clinical practice
ICR: The Institute of Cancer Research
ICR-CTSU: The Institute of Cancer Research – Clinical Trials and Statistics Unit
